# Structural basis for the activity regulation of *Medicago* calcium channel CNGC15

**DOI:** 10.1038/s41421-025-00815-y

**Published:** 2025-07-22

**Authors:** Xia Xu, Qinrui Wang, Tengfei Sun, Heyi Gao, Ruichu Gu, Junzhao Yang, Jiaqi Zhou, Peng Fu, Han Wen, Guanghui Yang

**Affiliations:** 1 Frontiers Science Center for Molecular Design Breeding, State Key Laboratory of Plant Environmental Resilience, College of Biological Sciences, China Agricultural University, Beijing, China; 2 DP technology, Beijing, China; 3 Beijing Advanced Center of RNA Biology (BEACON), Peking University, Beijing, China; 4 Institute for Advanced Algorithms Research, Shanghai, China; 5 AI for Science Institute, Beijing, China; 6 State Key Laboratory of Medical Proteomics, Beijing, China

**Keywords:** Cryoelectron microscopy, Plant signalling

## Abstract

Cyclic nucleotide-gated ion channels (CNGCs) in plants mediate Ca^2+^ influx in response to environmental changes. Among numerous plant CNGCs, *Medicago truncatula* CNGC15a/b/c (*Mt*CNGC15) is localized to the nuclear envelope. The opening and closing cycle of *Mt*CNGC15 is tightly associated with the Ca^2+^ oscillation in symbiosis. However, the molecular mechanism underlying *Mt*CNGC15 activity regulation remains unclear. In this study, we present the structures of *Mt*CNGC15 in its apo form and in the presence of CaM. The apo *Mt*CNGC15b exhibits a flexible cytoplasmic domain (CPD), whereas binding of the *Mt*CaM inhibits Ca^2+^ currents and stabilizes the highly dynamic CPD. Furthermore, the activity of *Mt*CNGC15b seems to be independent of cGMP. The hypothetical binding pocket for cGMP is occupied by an arginine residue. These findings elucidate the structural basis for the activity regulation of nuclear localized *Mt*CNGC15.

## Introduction

In plant cells, intracellular Ca^2+^ levels fluctuate rapidly in response to environmental stresses. The basal concentration of intracellular Ca^2+^ is approximately 100 nM, whereas the extracellular or nuclear envelop Ca^2+^ can reach to the millimolar level^1^. Activation of calcium channels or transporters is an prerequisite event to change the cellular Ca^2+^ concentration when plants sense different stimulus^2^. For instance, the binding of Nod factors to receptor kinases localized on the root cell membrane will trigger downstream Ca^2+^ changes for nodulation and symbiosis. A conserved sustained and rhythmic changes in nuclear Ca^2+^ concentration, termed as Ca^2+^ oscillations, plays a central role in regulating the transcription of genes required for nodulation and symbiosis^3,4^. Depiction of the key components in governing such process led to the identification of nuclear membrane specifically localized Ca^2+^ channels CNGC15a/b/c in *Medicago truncatula*^5^. The *Mt*CNGC15 was reported to be activated after the sensing of Nod factors and negatively regulated by camodulin (CaM) to fullfil a cycle of Ca^2+^ release and retrieve between the endoplasmic reticulum/nuclear envelop lumen and the nucleoplasm^6^. In addition, other Ca^2+^ transporters, such as the Ca^2+^ ATPase MCA8, further facilitate the Ca^2+^ recycling^1,2^.

The *Mt*CNGC15a/b/c belongs to the superfamily of CNG ion channels, which are widely distributed from prokaryotic to eukaryotic species^7^. These channels mediate the permeation of various cations, including Ca^2+^, Na^+^ and K^+^. In animals, CNG ion channels assemble either as homo-tetramers such as TAX-4 in *Caenorhabditis elegans*, CNGA1 in human, or hetero-tetramers composed of different subunits in cone and rod cells^8,9^. In *Arabidopsis,* 20 homologs of CNG ion channels (CNGC1-20) participate in distinct signaling pathways^10^. Among the CNGCs identified in *M. truncatula*, CNGC15a/b/c is specifically localized to nuclear membrane. However, some remaining questions restrict the rational regulation of the Ca^2+^ oscillations. First, how the structure of *Mt*CNGC15 changes upon CaM binding needs to be elucidated. Second, whether *Mt*CNGC15 can be gated by cyclic nucleotide monophosphate (cNMP) is not examined before. Although *Mt*CNGC15 has a probable cNMP binding domain (CNBD) and the *Arabidopsis* CNGCs have been reported to be affected by cNMP^11,12^, structural evidence remains lacking.

Here, we report the structures of *Mt*CNGC15b in the absence or presence of CaM. Binding of CaM may stabilize the conformation of *Mt*CNGC15b cytoplasmic domain to modulate the channel open and close cycle. Different from the several reported animal CNG channels, we prove that *Mt*CNGC15b is not gated by cNMP. Instead, the Arg553 of *Mt*CNGC15b occupies the corresponding cNMP pocket in animal homologs. These findings provide structural and functional insight into *Mt*CNGCs activity regulation in symbiosis.

## Results

### Architecture of *Mt*CNGC15b

**Fig. 1:**
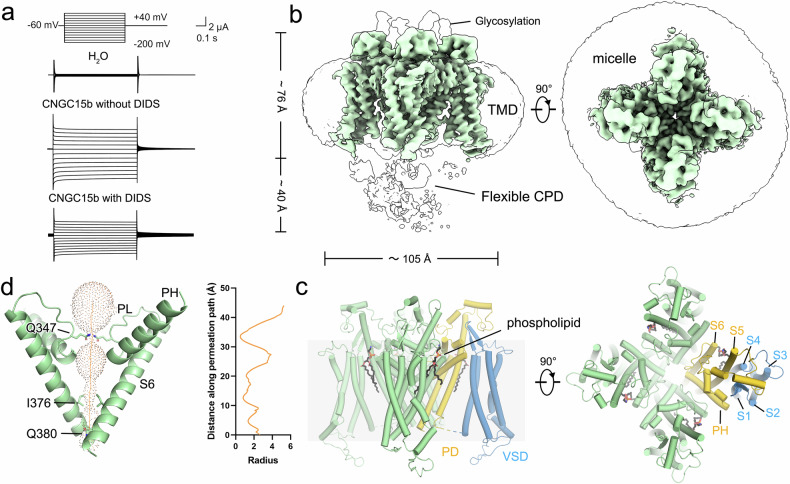
Architecture of the *Medicago truncatula* calcium channel *Mt*CNGC15b. **a** TEVC recording from *Xenopus* oocytes expressing *Mt*CNGC15b. In Ca^2+^-containing bath solution buffer, 500 μM DIDS was added to inhibit the activity of the Ca^2+^-activated chloride channel. **b** Density map of *Mt*CNGC15b with the clear transmembrane domain and the flexible cytoplasmic domain. **c** Architecture of the transmembrane region of *Mt*CNGC15b channel. **d** Pore radius of *Mt*CNGC15b calculated by MOLE^39^.

To explicate the structure of *Mt*CNGC15, the expression level and cryo-EM performance of three isoforms *Mt*CNGC15a/b/c were individually examined using insect cells and mammalian cells. In spite of high sequence similarities, the *Mt*CNGC15b exhibits relatively higher expression level and better protein purity in mammalian expression system. Since *Mt*CNGC15b is localized to the nuclear membrane, we constructed the hemagglutinin (HA) signal peptide (MKTIIALSYIFCLVFA) to the N-terminus of *Mt*CNGC15b to aid their localization to the cell membrane^13^. In the two-electrode voltage clamp assay, *Mt*CNGC15b demonstrated robust Ca²^+^ currents (Fig. 1a). In the following cryo-EM analysis, 2D classification indicated that the cytoplasmic domains (CPDs) of *Mt*CNGC15b are highly flexible (Supplementary Fig. S1a; Fig. 1b).

The transmembrane domains (TM) and CPD of the *Mt*CNGC15b show contrasting features in the density map. The well-resolved EM density of TMs enabled the unambiguous assignment of residues 58-385 from *Mt*CNGC15b (Fig. 1c; Supplementary Fig. S1b and Table S1). The TMs of *Mt*CNGC15b channel is ~76 Å in height and ~105 Å in width when viewed perpendicular to the membrane neighboring subunits (Fig. 1b). Similar to other CNG channels, the transmembrane domains of *Mt*CNGC15b can be divided into the voltage sensing domain (VSD, S1–S4), the pore domain (PD, S5–S6), and the selectivity filter (SF) (Fig. 1c, d). The ion conduction pathway of *Mt*CNGC15b consists of the selectivity filter (SF) in pore loops and the inner gate formed by S6 (Fig. 1d).

### The selectivity filter of *Mt*CNGC15b for recognition of Ca^2+^

**Fig. 2:**
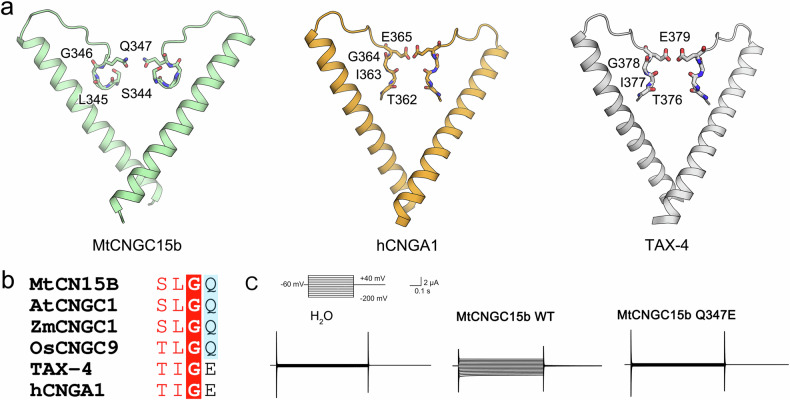
The selectivity filter of *Mt*CNGC15b. **a** Comparison of S6 and SF of *Mt*CNGC15b to TAX-4 (PDB: 6WEJ) and human CNGA1 (PDB: 7LFT)^8,9^. **b** Sequence alignment of the selectivity filter between representative plant and animal CNG channels. The Gln residue displays high conservation in plant CNGCs. **c** Substitution of Gln347 to Glu abrogates the *Mt*CNGC15b-mediated Ca^2+^ currents.

The SF of MtCNGC15b comprises residues Ser344-Gln347 (Fig. 2). The side chains of Gln347 restrict the permeation path, differing from a glutamic acid in reported animal CNG ion channels like TAX-4 and human CNGA1 (Fig. 2a). Sequence alignment with three other CNGCs from *Arabidopsis*, *Oryza sativa*, and *Zea mays* reveals the conservation of Gln residue in plant CNG channels (Fig. 2b; Supplementary Fig. S2). Substitution of Gln347 to Glu abolished the Ca^2+^ currents, suggesting the indispensable role of Gln347 in Ca^2+^ recognition (Fig. 2c). By contrast, both TAX-4 and human CNGA1 have relatively broad range of ion selectivity^8,9^. To investigate the ion conductivity of *Mt*CNGC15b and the Q347E variant towards Na^+^ and K^+^, we detected the reversal potentials and calculated the ion permeability ratios (Supplementary Table S2)^14^. Both *Mt*CNGC15b and Q347E are localized on the cell membrane and exhibit low conductivity of K^+^ (Supplementary Fig. S3 and Table S2). Based on the ion permeability ratios, *Mt*CNGC15b WT exhibits nearly equal permeability to calcium and sodium ions (*P*_Ca_/*P*_Na_: ~1.03) but demonstrates higher selectivity for Ca^2+^ and Na^+^ over K^+^ (*P*_K_/*P*_Ca_: ~0.183, *P*_Na_/*P*_K_: ~9.44). By contrast, the *P*_Ca_/*P*_Na_ reduced to ~0.164 for the Q347E mutation, indicating a qualitative change of Ca^2+^ selectivity. Although the mutation of Q347E exhibits low ion conductivity towards all the three ions, its permeation ration reflects higher Na^+^ selectivity over Ca^2+^ and K^+^ (Supplementary Table S2).

**Fig. 3:**
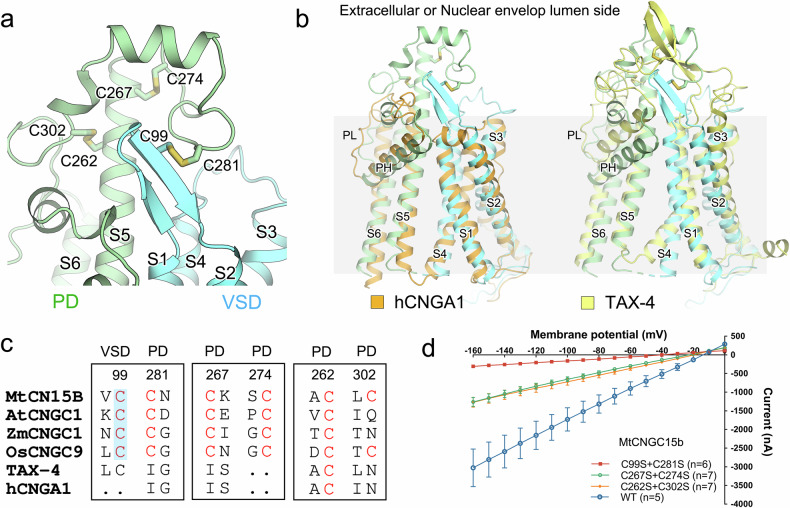
The coordination between VSD and PD of *Mt*CNGC15b. **a** Structural differences between *Mt*CNGC15b with human CNGA1 or *C. elegans* TAX-4. **b** Three pairs of disulfide bonds are located at the nuclear envelop lumen side. The Cys99-Cys281 mediated the interaction between VSD and PD. **c** Sequence and structural comparison of the extracellular cysteine pairs in representative plant CNGCs and animal CNG channels. The Cys99-Cys281 mediated-disulfide bond is specifically observed in plant CNGCs. **d** Disruption of the identified three disulfide bonds decreases the Ca^2+^ currents. Particularly, mutations of the Cys99-Cys281 pair nearly abolish the channel activity. Current–voltage relationship of *Mt*CNGC15b and its variants currents recorded from *Xenopus* oocytes. Command voltages were applied in 10-mV steps between 0 mV and –160 mV. Data are presented as means ± SEM.

Different from animal CNG channels, three pairs of disulfide bonds — Cys99/Cys281, Cys262/Cys302, and Cys267/Cys274 on *Mt*CNGC15b is mapped to the nuclear envelop lumen side (Fig. 3a, b). Notably, the Cys99/Cys281 pair forms a bridge between the VSD and the PD of *Mt*CNGC15b. Such structural element is conserved among plant CNG channels but not TAX-4 or human CNGA1 (Fig. 3c). Given that the lumenal environment is likely oxidative, these disulfide bonds may play a physiological role in regulating the coupling between the VSD and PD. Mutation of Cys99 and Cys281 to Ser drastically reduced Ca^2+^ currents without altering the localization of *Mt*CNGC15b in the plasma membrane, while mutations in the other two cysteine pairs also led to compromised activity (Fig. 3d; Supplementary Fig. S3).

### *Mt*CaM negatively regulates *Mt*CNGC15b activity by modulating CPD conformation

**Fig. 4:**
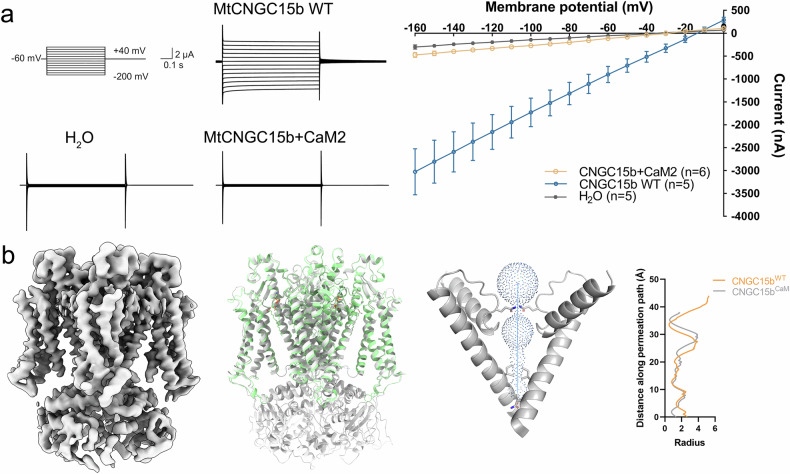
*Mt*CaM stabilizes the CPD of *Mt*CNGC15b and closes the channel. **a** TEVC recording from *Xenopus* oocytes expressing *Mt*CNGC15b and *Mt*CaM. In Ca^2+^-containing bath solution buffer, 500 μM DIDS was added to inhibit the activity of the Ca^2+^-activated chloride channel. Command voltages were applied in 10-mV steps between 0 mV and –160 mV. The *I*–*V* curves were obtained from the peak currents at command voltages. Data are presented as means ± SEM. **b** The CPD of *Mt*CNGC15b can be stabilized by CaM. The inner gate region of *Mt*CNGC15b is regulated upon involvement of CaM. Pore radius is calculated by MOLE^39^.

*Mt*CaM has been reported to negatively regulate the activity of *Mt*CNGC15b (Fig. 4a), but how *Mt*CaM binding affects the CPD of *Mt*CNGC15b remains to be elucidated. To unravel the probable molecular changes, we tried to co-express *Mt*CaM and *Mt*CNGC15b for cryo-EM structural study. However, the *Mt*CaM was not stably co-purified with *Mt*CNGC15b. We then fused the *Mt*CaM to the C-terminus of *Mt*CNGC15b through a short linker. The fused complex exhibited good solution behavior and dispersed evenly on cryo-EM samples. Although the cryo-EM densities of *Mt*CaM itself were invisible, we can observe the density of the *Mt*CNGC15b CPD (Fig. 4b; Supplementary Fig. S3). Consistent with the previous findings on the inhibitory effect on channel activity^15^, *Mt*CNGC15b exhibits closed inner gate (Fig. 4b, right panel). To evaluate the impact of *Mt*CaM, we have employed AlphaFold 3^16^ to predict the potential binding site of *Mt*CaM and analyzed the residues at the interaction interface (Supplementary Fig. S5a). To validate these probable binding site and its regulatory effects, we introduced mutations on *Mt*CNGC15b and recorded the currents mediated by these variants (Supplementary Fig. S5b). Compared to wild-type *Mt*CNGC15b, point mutations compromised the inhibitory effect of *Mt*CaM, leading to weak recovery of currents.

### The putative cNMP binding pocket of *Mt*CNGC15b is occupied by an arginine

**Fig. 5:**
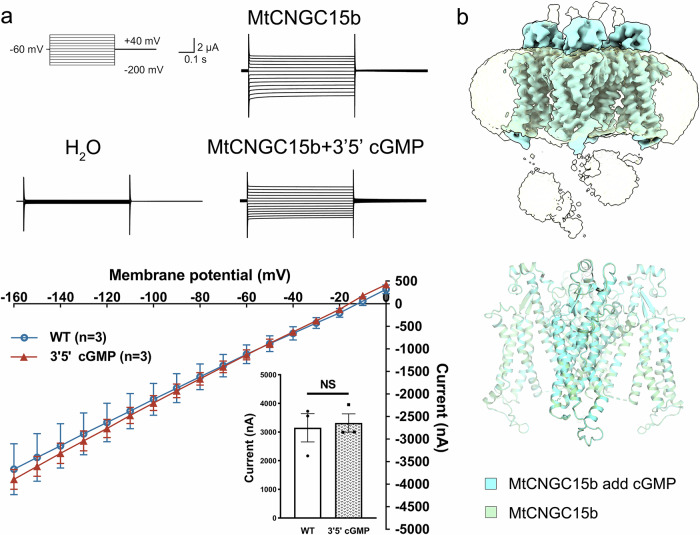
*Mt*CNGC15b is not gated by cGMP. **a** TEVC recording from *Xenopus* oocytes expressing *Mt*CNGC15b and add 5 mM 3′5′ cGMP. In Ca^2+^-containing bath solution buffer, 500 μM DIDS was added to inhibit the activity of the Ca^2+^-activated chloride channel. The *I*–*V* curves were obtained from the peak currents at command voltages. The currents had no significant difference in the absence or presence of cGMP (inset panel). Current amplitudes at –140 mV from multiple recordings. Data are presented as means ± SEM, *n* = 3 biologically independent oocytes. **b** Structrual superimposition of *Mt*CNGC15b in the presence of 3′5′ cGMP with the apo state. Addition of 3′5′ cGMP results in an identical density map with highly flexible CPD.

The CNG channels are widely reported to be gated by cGMP or cAMP in animals. Previous studies on plant CNGCs suggest that cyclic nucleotides (cNMPs) influence CNGC activity, which seems plausible given that plant CNGCs possess a predicted cyclic nucleotide-binding domain (CNBD) in their structures^17^. Based on these assumptions, we sought to validate whether cGMP binds to *Mt*CNGC15b. First, we assessed the binding affinity of cGMP/cAMP to *Mt*CNGC15b through surface plasmon resonance. The results show that *Mt*CNGC15b has negligible binding capacity for cGMP/cAMP (Supplementary Fig. S6). Second, cGMP treatment has no impact on Ca^2+^ currents (Fig. 5a). To further validate such observations, we supplemented 5 mM cGMP to *Mt*CNGC15b when preparing cryo-EM sample as described^12^. However, addition of cGMP reveals no structural changes. We observed flexible CPDs after 2D classification and even identical conformation of the transmembrane domains (Fig. 5b). Taken together, cNMP is not the ligand of *Mt*CNGC15b.

**Fig. 6:**
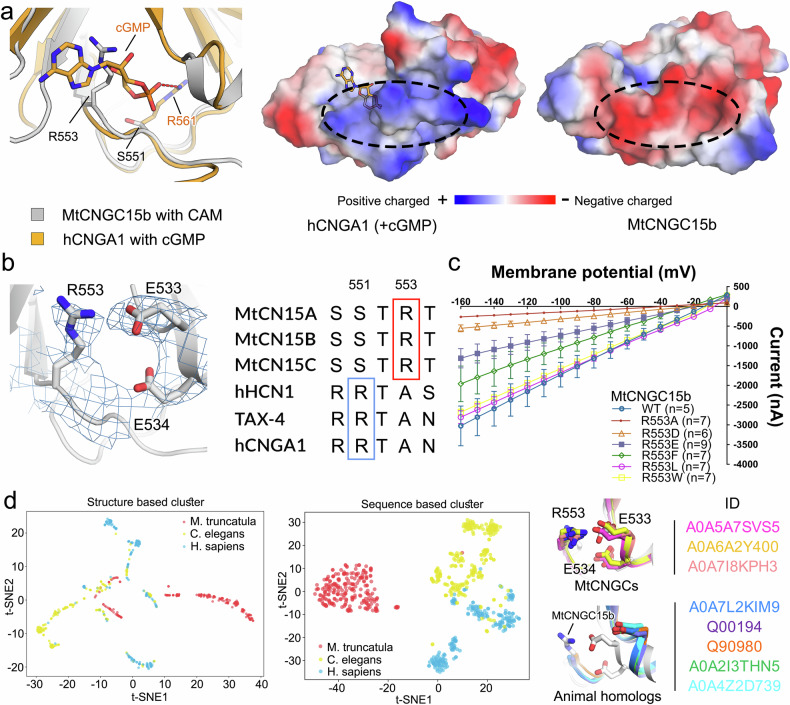
Arg553 occupies the putative cNMP pocket for *Mt*CNGC15b. **a** Structural alignment between the CNBD of *Mt*CNGC15b and the cGMP-bound human CNGA1. The corresponding region of the cGMP binding site in *Mt*CNGC15b is negatively charged in comparison with human CNGA1. **b** The basic side chain of Arg553 is compatible with the negatively charged pocket of *Mt*CNGC15b composed by Glu533 and Glu534. The Arg553 is conserved in *Medicago* but not representative animal CNG channels. **c** Mutagenesis of Arg553 to different amino acids decreases the Ca^2+^ currents. Current-voltage relationship of *Mt*CNGC15b and its variants currents recorded from *Xenopus* oocytes. Command voltages were applied in 10-mV steps between 0 mV and –160 mV. Data are presented as means ± SEM. **d** Sequence and structure clustering results of 726 sequences that share over 80% similarity with CNG channels from *C. elegans*, *H. sapiens*, and *M. truncatula*. The representative predicted structures are aligned to *Mt*CNGC15b with the entries number listed.

We then superimposed the *Mt*CNGC15b CNBD with human CNGA1 bound with cGMP. In human CNGA1, the cGMP phosphor group is nestled into a positively charged pocket and coordinated by the side chain of Arg561. This arginine is absence and aligned to a serine residue in *Mt*CNGC15b. Moreover, the putative cGMP binding pocket in *Mt*CNGC15b is negatively charged (Fig. 6a). Instead of accommodating cGMP, the side chain of Arg553 in *Mt*CNGC15b occupies the hypothetical pocket. The basic nature of Arg553 is compatible with the negatively charged pocket in *Mt*CNGC15b (Fig. 6a, b). Sequence alignment reveals the cNMP-binding arginine is conserved in representative animal cNMP-gated channels, whereas in plant CNGCs, an arginine residues occupies this position (Fig. 6b; Supplementary Fig. S7). Substituting Arg553 by several kinds of residues decreased the Ca^2+^ influx in varing degrees, while replacement of the arginine residue with alanine nearly abolished Ca^2+^ currents. The results showed that the R553L and R553W mutations did not significantly reduce ion channel activity, suggesting that changes in amino acid polarity have a limited impact. Instead, side chain size appears to play a more critical role in regulating channel function (Fig. 6c). Such structural element reminiscent of that the activity regulation by intrinsic ligand in CNBD-like domains of a zebrafish channel, zKCNH. In zKCNH, a C-terminal short β-strand occupied the presumable cNMP binding pocket^18^.

To investigate potential evolutionary differences associated with the presence or absence of this arginine residue, we employed the ESM2-150M model to generate high-dimensional embeddings for 726 sequences derived from BLAST results in the UniProt database, using *C. elegans*, *H. sapiens*, and *M. truncatula* CNG channels as templates for sequences with high similarity. Among them, we utilized 505 available structures from the AlphaFold database to calculate pairwise structural similarities^19^. Both sequence and structure clustering results suggest that *M. truncatula* has followed a distinct evolutionary trajectory, potentially as an adaptation to environmental stress (Fig. 6d). As the potential intrinsic ligand observed in *Mt*CNGC15b is conserved across all aligned plants CNGCs except for *Arabidopsis thaliana At*CNGC2 and *At*CNGC4, we postulate that the plants CNGCs are evolved differently from animal ones.

Comparative analysis of CNBD structures reveals consistent residues in the local environment of R553 in *M. truncatula*, characterized by one arginine and two glutamates at equivalent positions of R553, E533 and E534. In contrast, while the overall conformations of the CNBD domains in *C. elegans* and *H. sapiens* resemble those in *M. truncatula*, the local interactions between arginine and glutamates are not preserved. This discrepancy may suggest that, in an immobile state, plants need to develop mechanisms for rapid responses to environmental stress. One such mechanism could involve the “ready-to-open” of certain ion channels through occupying the “presumably ligand pocket” by this arginine but not cNMP.

### *Mt*CaM binding may regulate the local environment near the arginine

**Fig. 7:**
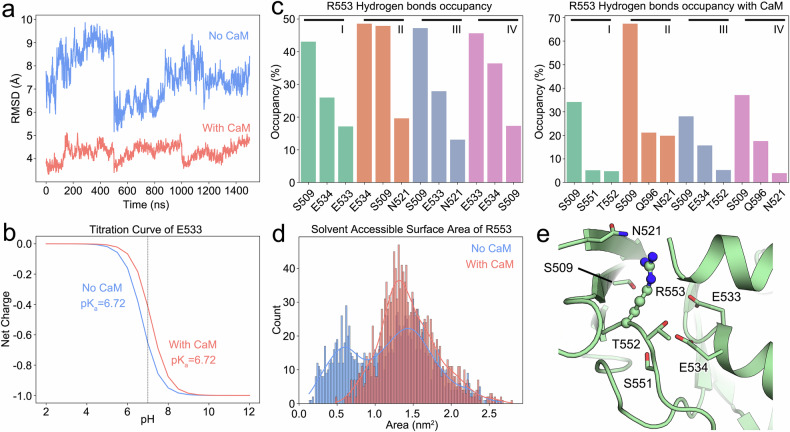
Molecular dynamic simulation of CNBD in the presence of *Mt*CaM. **a** RMSD of *Mt*CNGC15b after least square fit to backbone throughout the last 500 ns of three repeated simulations. **b** Calculated titration curve of E533 in *Mt*CNGC15b with or without CaM binding. Predicted pKa’s are indicated. **c** Three residues with the highest hydrogen bonding occupancy with R553 in each chain throughout the last 500 ns of three repeated simulations in the absence or presence of CaM. **d** Distribution of solvent accessible surface area of R553 with or without *Mt*CaM binding to *Mt*CNGC15b throughout the last 500 ns of three repeated simulations. **e** Local environment of Arg553.

To gain insights into whether involvement of *Mt*CaM has influence on the regulation of this arginine residue, we conducted molecular dynamics (MD) simulation of the CPD of *Mt*CNGC15b in the absence or presence of *Mt*CaM. *Mt*CaM binding significantly stabilizes the cytosolic domain of *Mt*CNGC15b, as evidenced by a lower RMSD and confirmed by the structural observation (Fig. 7a). Upon *Mt*CaM binding, the local environment around Glu533 changes compared to its state without *Mt*CaM. At pH 7.0, Glu533 becomes protonated in the presence of *Mt*CaM, whereas it remains negatively charged without *Mt*CaM (Fig. 7b). This protonation reduces the interaction between Glu533, Glu534, and Arg553 (Fig. 7c). The solvent-accessible surface area (SASA) of Arg553 and Glu533 further suggests that CaM binding disrupts their interaction, making these residues more solvent-exposed (Fig. 7d). Notably, Ser509 persistently interacts with R553 regardless the presence of CaM (Fig. 7c–e). To further validate these computational data, we performed electrophysiological experiments on E533Q and S509A. Within expectation, both mutations severely compromised the Ca^2+^ currents, indicating their important role in regulating the channel activity (Supplementary Fig. S8).

## Discussion

During the symbiosis, *Mt*CNGC15b is considered as a Ca^2+^ channel for Ca^2+^ oscillation and regulated by camodulin. The current observation that *Mt*CNGC15b has an intrinsic-ligand-like arginine residue indicates that *Mt*CNGC15b channels have evolved to function without cNMP regulation. The presence of Arg553 and other unique residues reflects a natural result of their evolutionary trajectory, rather than a targeted adaptation to substitute for cNMP regulation. Such hypothesis might be true in other plants CNGCs as well because there is no adenylate cyclase homologs identified in plants^20^. Our structural and functional evaluations on *Mt*CNGC15b are in agreement with the very overlapping findings on *Arabidopsis* CNGC1/5^21^, including the Ca^2+^ selectivity determination, the extracellular/luminal side disulfide bonds and the putative intrinsic ligand.

In addition, the CNBD domain exists widely in plant membrane proteins, exemplified by K^+^ channel AKT1 and Na^+^ exchanger SOS1^22,23^. These ion channel or transporters are regulated by phosphorylation, with most posttranslational modification sites located on the cytoplasmic domains. No ligands have been identified to bind the CNBD of AKT1 and SOS1. Similar to *Mt*CNGC15b, *At*CNGC1/5 and zebrafish KCNH, both AKT1 and SOS1 may exhibit an intrinsic-ligand-like mode of regulation (Supplementary Fig. S9). Such hypothesis is further supported by recent studies on the intrinsic ligand-like residues of *Arabidopsis* potassium channel GORK^21,24,25^.

Based on the structural observation and electrophysiological analysis, *Mt*CaM may close the channel through stabilizing the CPD of *Mt*CNGC15b and modulating the local conformation of residues near the arginine. These findings support the previously reported modulatory effect of the engineered mutant *Mt*CaM^R91A^. This mutation exhibits considerable flexibility, which may regulate the on-off rate of *Mt*CaM and thereby enhance the frequency of calcium oscillations^6^.

In conclusion, we report a distinct regulatory mode for the nuclear the nuclear membrane-localized *Mt*CNGC15b. We propose a regulation mode for *Mt*CNGC15b involving an intrinsic-ligand-like residue that occupies the pocket for cNMP. Such mechanism may sensitilize the channel in a “ready-to-open” state for the rapid response and Ca^2+^ oscillation in symbiosis. In other plant CNGCs, calmodulin binding has been shown to negatively regulate channel activity as well. Such negative feedback mode could desensitilize the channel to avoid hyper-responses. Our results lay the molecular basis for the structural-functional relationship of this channel and pave the way for further mechanistic studies on the functional regulation of plant CNGC channels.

## Materials and methods

### Purification of *M. truncatula* CNGC15b and CNGC15b-CaM

Full-length coding sequences of *M. truncatula* CNGC15b (Uniprot ID: G7JND3) were synthesized and subcloned into a pCAG vector with a C-terminal Flag-TwinStrep tag between *Kpn*I and *Xho*I restriction sites, respectively. The recombinant CNGC15b was expressed using mammalian cell (HEK293F) expression system. Briefly, a mixture of 1.5 mg CNGC15b encoding plasmids and 4 mg polyethylenimine (Polysciences) was added into 1 L HEK293F cells at a density of ~2.5 × 10^6^ cells mL^–1^. After transfection for 60 h under 37 °C with 5% CO_2_, cells were collected and homogenized in lysis buffer containing 25 mM HEPES pH 7.4, 150 mM NaCl supplemented with protease inhibitor cocktails consisting of 1 mM phenylmethylsulfonyl fluoride (PMSF), 2.6 μg mL^−1^ aprotinin, 2 μg mL^−1^ pepstatin and 4 μg mL^−1^ leupeptin.

Then cell membranes were solubilized with 1% (w/v) decyl maltose neopentyl glycol (DMNG, Anatrace) and 0.1% (w/v) cholesteryl hemisuccinate tris salt (CHS, Anatrace) at 4 °C for 2 h with gently agitation. After centrifugation at 45,000 rpm for 30 min, the supernatant was applied to anti-Flag M2 affinity gel (Sigma) through gravity column (Bio-rad). The affinity gel was rinsed with wash buffer containing 25 mM HEPES pH 7.4, 150 mM NaCl, 0.02% (w/v) GDN supplemented with protease inhibitor cocktails consisting of 1.3 μg mL^−1^ aprotinin, 1 μg mL^−1^ pepstatin and 2 μg mL^−1^ leupeptin for three times. Protein was eluted with wash buffer plus 0.2 mg mL^−1^ flag peptide (DYKDDDDK, synthesized by GL Biochem). Then the eluent was loaded onto *Strep*-Tactin (Smart-lifesciences) affinity column. After being rinsed with wash buffer containing 25 mM Tris pH 8.0, 150 mM NaCl, 0.02% (w/v) GDN, target protein was eluted with wash buffer plus 2.5 mM desthiobiotin. Then eluted protein was immediately concentrated with a 100 kDa cut-off concentrator (Millipore) and further purified by size-exclusion chromatography (SEC) using Superose 6 Increase 10/300 GL column (GE Healthcare) with SEC buffer containing 25 mM Tris pH 8.0, 150 mM NaCl, 0.006% (w/v) GDN. Peak fractions were pooled together and concentrated to ~10 mg mL^–1^ for cryo-EM sample preparation. The *Mt*CNGC15b that fused *Mt*CaM2 (Medtr5g088320) by a -GGS-x6 linker was purified following the same protocol.

### Cryo-EM sample preparation and data acquisition

Quantifoil 300 mesh R1.2/1.3 Au grids were glow discharged. Four microliters aliquots were applied to grids with Vitrobot Mark IV (Thermo Fisher Scientific) at 8 °C and 100% humidity. After blotted for 3 s, grids were flash-frozen in liquid ethane and transferred to liquid nitrogen for storage. The samples were screened on 200 kV Tecnai Arctica microscope, and grids with high quality were used for data acquisition. High-resolution data was collected on 300 kV Titan Krios microscope equipped with K3 Summit electron detector (Gatan) and a GIF Quantum energy filter (slit width 20 eV, Gatan) at super-resolution counting mode. A series of defocus values from –1.3 to –1.8 μm was used during data collection. Each image was dose-fractionated to 32 frames with a total electron dose of about 50 e^−^Å^−2^ and a total exposure time of 2.56 s. EPU software (Thermo Fisher) was used for data collection.

### Data processing

For the cryo-EM analysis of *Mt*CNGC15b, *Mt*CNGC15b-CaM, movie stacks have been collected and processed following the tutorial of CryoSPARC^26^. The average resolution of *Mt*CNGC15b or *Mt*CNGC15b-CaM is 3.1 Å or 3.6 Å on the basis of the Fourier shell correlation (FSC) 0.143 criterion. The FSC curves were corrected for the effects of a soft mask using high-resolution noise substitution. Local resolution variations were estimated using CryoSPARC^26^.

### Model building, refinement and validation

The models were manually adjusted based on the predicted model from AlphaFold2 database^19,27^. The structure was then refined in real space using PHENIX with secondary structure and geometry restraints^28^. The atomic model was manually improved using COOT^29^. The final atomic models were refined in real space using PHENIX. The final atomic model was evaluated using MolProbity^30^.

### Two-electrode voltage clamping recording from *Xenopus* oocytes

The cDNAs for *Mt*CNGC15b, *Mt*CNGC15b variants and *Mt*CaM2 were cloned into the pGEMHE oocyte expression vector. Since *Mt*CNGC15b is localized to the nuclear membrane, we fused the hemagglutinin (HA) signal peptide (MKTIIALSYIFCLVFA) to the N-terminus of all *Mt*CNGC15b and its variants to aid their localization to the cell membrane^13^. For making HA-*Mt*CNGC15b-EGFP fusion, the EGFP tag was amplified by PCR and inserted in-frame to the C terminus of the *Mt*CNGC15b into pGEMHE-HA-*Mt*CNGC15b. Transcription and capped RNAs (cRNAs) were synthesized from 2 μg of linearized plasmid DNA template using the T7 High Yield RNA Transcription Kit and Vaccinia Capping Enzyme Kit (Vazyme), according to the manufacturer’s recommendations. The quality of the cRNA was checked by agarose gel electrophoresis. 50 ng of each cRNA was injected into each oocyte. For recording of CNGC15b + CaM2, a 50 ng-mixture of two cRNAs (25 ng for CNGC15b and 25 ng for CaM2) was injected into one oocyte. Injected oocytes were incubated in ND96 solution (96 mM NaCl, 2 mM KCl, 1 mM MgCl_2,_ 1.8 mM CaCl_2_, 10 mM HEPES/NaOH, pH 7.4) supplemented with 0.1 mg/mL gentamycin and streptomycin at 18 °C for 2 days before electrophysiological recording. Oocytes were voltage-clamped using a Axoclamp 900 A amplifier (Axon Instruments) and monitored by computer through Digidata 1440 A converter (Axon CNS) and Clampex 11.2 software (Axon Instruments). The pipette solution contained 3 M KCl. The oocytes were continuously perfused during the voltage-clamp experiment. The standard bath solution contained 5 mM CaCl_2_, 2 mM KCl, 1 mM MgCl_2_ and 10 mM MES/Tris (pH 5.6). Moreover, 500 μM DIDS was added to inhibit the activity of the Ca^2+^-activated chloride channel. The osmolalities of all solutions were adjusted to 220 mOsmol kg^–1^ using mannitol. Voltage steps were applied from +40 to –200 mV in –10/–20 mV decrements during 0.8 s, from a holding potential of –60 mV. Each step begins with 0.03 s and ends with 1.4 s at the resting potential of the oocyte membrane in the tested bath solution. For recording of CNGC15b + 3′5′ cGMP, 50 nL of 5 mM 3′5′ cGMP was injected into one oocyte which has already been injected with CNGC15b cRNA 2 days ago, and then incubated in ND96 solution at 18 °C for 1 h before TEVC experiment.

### Sequence-based clustering and structure-based clustering

The ESM2-150M model was utilized to generate high-dimensional representations of each original sequence. Subsequently, we employ *t*-SNE method to reduce these representations to two dimensions for visualization. Structural data for the sequences were obtained from AlphaFold database, yielding structures for 505 of the 726 original sequences. We calculated pairwise structural similarity using the tmscoring package, determining the TM-score between structures and defining the structural distance as (1 − TM-score). Through this approach we generated a 505 × 505-dimensional distance matrix. The *t*-SNE method was applied to reduce these structural representations to two dimensions for visualization.

### Molecular dynamics simulation

*Mt*CNGC15b structures with or without calmodulin were modeled with AlphaFold3. Considering the region of interest, the cytosolic domain of *Mt*CNGC15b (T387-C620) was retained. The systems were solvated in 0.15 M NaCl solution. GROMACS^31^ version 2024.2 with the CHARMM36 force field^32^ and TIP3P water model^33^ were used to perform the simulations. During NVT, NPT equilibrations, position restraints on protein heavy atoms were applied with a force constant of 1000 kJ/mol nm^2^. To minimize the impact of the absence of the transmembrane domain, a position restraint with force constant of 1000 kJ/mol nm^2^ was applied to the backbone atoms of the C-linter region (T387-T428) throughout the entire simulations. Three repeats of 700 ns atomistic production simulations were then performed for each system.

The long-range electrostatics (< 1 nm) was modeled using the Particle Mesh Ewald (PME) method^34^. The first step of equilibration was conducted under an NVT ensemble for 125 ps with integration time step of 1 fs, followed by an NPT equilibration phase with the same integration time step for 125 ps. Berendsen thermostat^35^ with a reference temperature of 301.15 K was used for temperature coupling during the entire equilibration phase. Berendsen barostat^35^ with a reference pressure of 1 bat and a compressibility of 4.5 × 10^−5^/bar was applied for pressure control during NPT equilibration. For production runs, v-rescale thermostat^36^ and Parrinello-Rahman barostat^37^ were used for temperature and pressure coupling, respectively. Covalent bonds are constrained to their equilibrium length by the LINCS algorithm^38^ for all simulations.

Radius of gyration (ROG) was calculated using MDAnalysis (https://docs.mdanalysis.org/2.7.0/index.html). Since the tetrameric structure was aligned with respect to the Z-axis, the ROG essentially describes the average distance between each site to the center of the protein in *X*–*Y* plane. Based on the equilibrium MD simulations of CNGC with and without calmodulin (between 200 and 700 ns), we averaged ROG over three MD trajectories, and then computed the change of average ROG (n) from without to with calmodulin to quantify the calmodulin-induced expansion/contraction at residue position n.

## Supplementary information

Supplementary information, Figures and Tables

## Data Availability

The cryo-EM maps of the whole protein and the transmembrane domain of *Mt*CNGC15b and the *Mt*CNGC15b-CaM complex have been deposited in the Electron Microscopy Data Bank (EMDB) with the accession code EMD-62261, EMD-62262. The atomic coordinates for the corresponding model have been deposited in the Protein Data Bank (PDB) under the accession code 9KCU, 9KCV, respectively.

## Materials availability

Requests for materials should be addressed to G.Y. (guanghuiyang@cau.edu.cn).
