# Supplementary material for: Structural basis for the activity regulation of Medicago calcium channel CNGC15
Source: Cell Discov. 2025 Jul 22;11:63. doi: 10.1038/s41421-025-00815-y (PMC12284214; doi:10.1038/s41421-025-00815-y)
Supplement: Supplementary file 1 — Supplementary information, Figures and Tables [file 41421_2025_815_MOESM1_ESM.pdf]

Supplementary information for

Structural basis for the activity regulation of *Medicago* calcium channel CNGC15

Xia Xu[1*], Qinrui Wang[2*], Tengfei Sun[1], Heyi Gao[1], Ruichu Gu[2], Junzhao Yang[1], Jiaqi Zhou[1], Peng Fu[1], Han Wen[2,3,4,5,6,7], Guanghui Yang[1,7]

[1]Frontiers Science Center for Molecular Design Breeding, State Key Laboratory of Plant Environmental Resilience, College of Biological Sciences, China Agricultural University, Beijing 100193, China

[2]DP technology, Beijing 100190, China

[3]Beijing Advanced Center of RNA Biology (BEACON), Peking University, Beijing, 100871, China.

[4]Institute for Advanced Algorithms Research, Shanghai, 201799, China.

[5]AI for Science Institute, Beijing, 100085, China.

[6]State Key Laboratory of Medical Proteomics, China

[*]These authors contribute equally to this work.

[7]To whom correspondence should be addressed. E-mail: wenh@dp.tech; guanghuiyang@cau.edu.cn

The file includes:
Supplementary Figures S1 to S9
Supplementary Table S1, S2

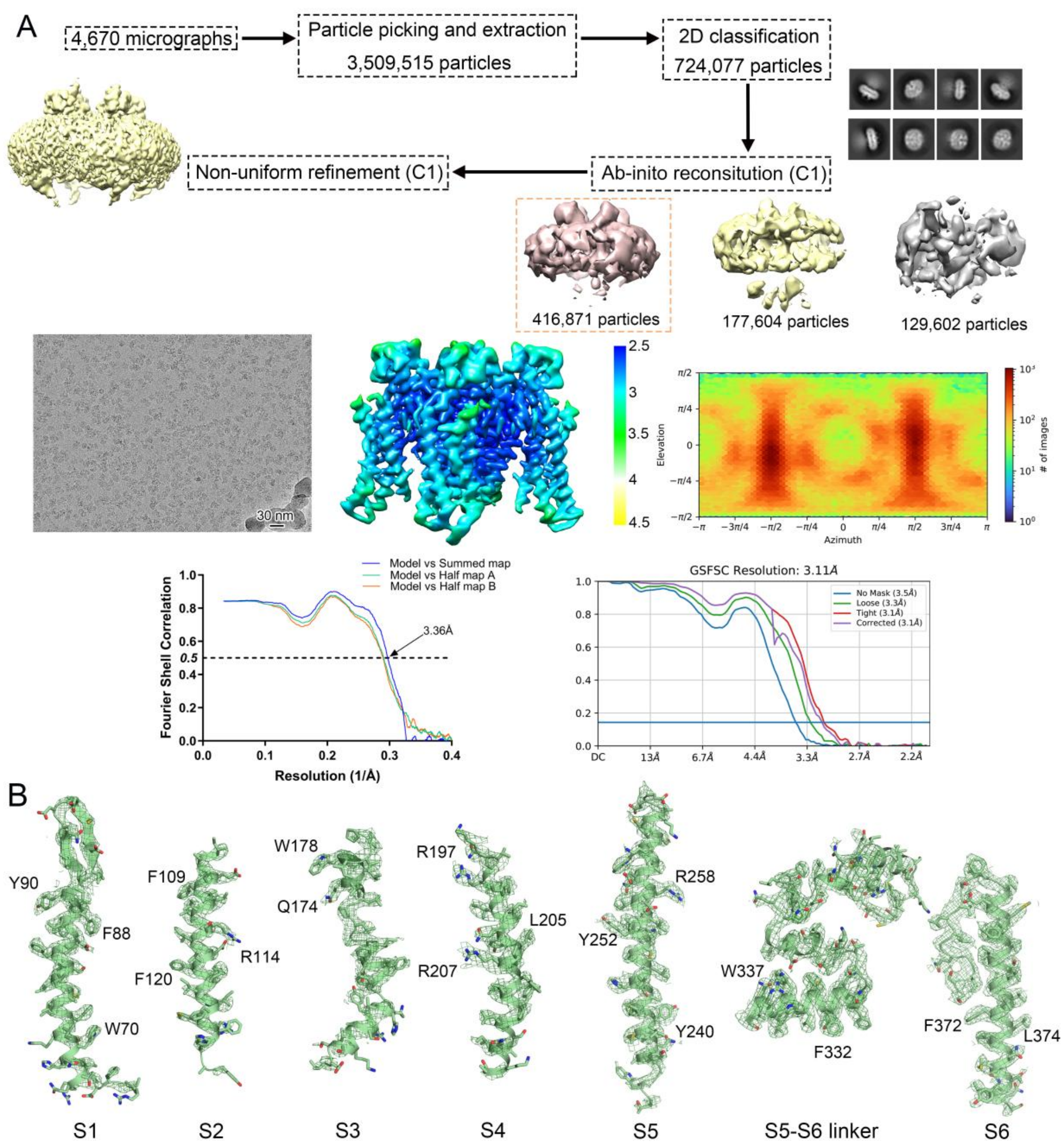

Supplementary Fig. 1 | Cryo-EM analysis of *Mt*CNGC15b. **a,** Data processing for *Mt*CNGC15b. The average resolution for the final reconstruction of *Mt*CNGC15b is estimated to be 3.1 Å. Details are described in the Methods. Representative micrograph and 2D classification results are shown. Local resolution distribution of the final reconstruction for *Mt*CNGC15b is estimated by cryoSPARC[28]. **b,** The local EM density map for *Mt*CNGC15b. The contour level of the EM density is 7σ.

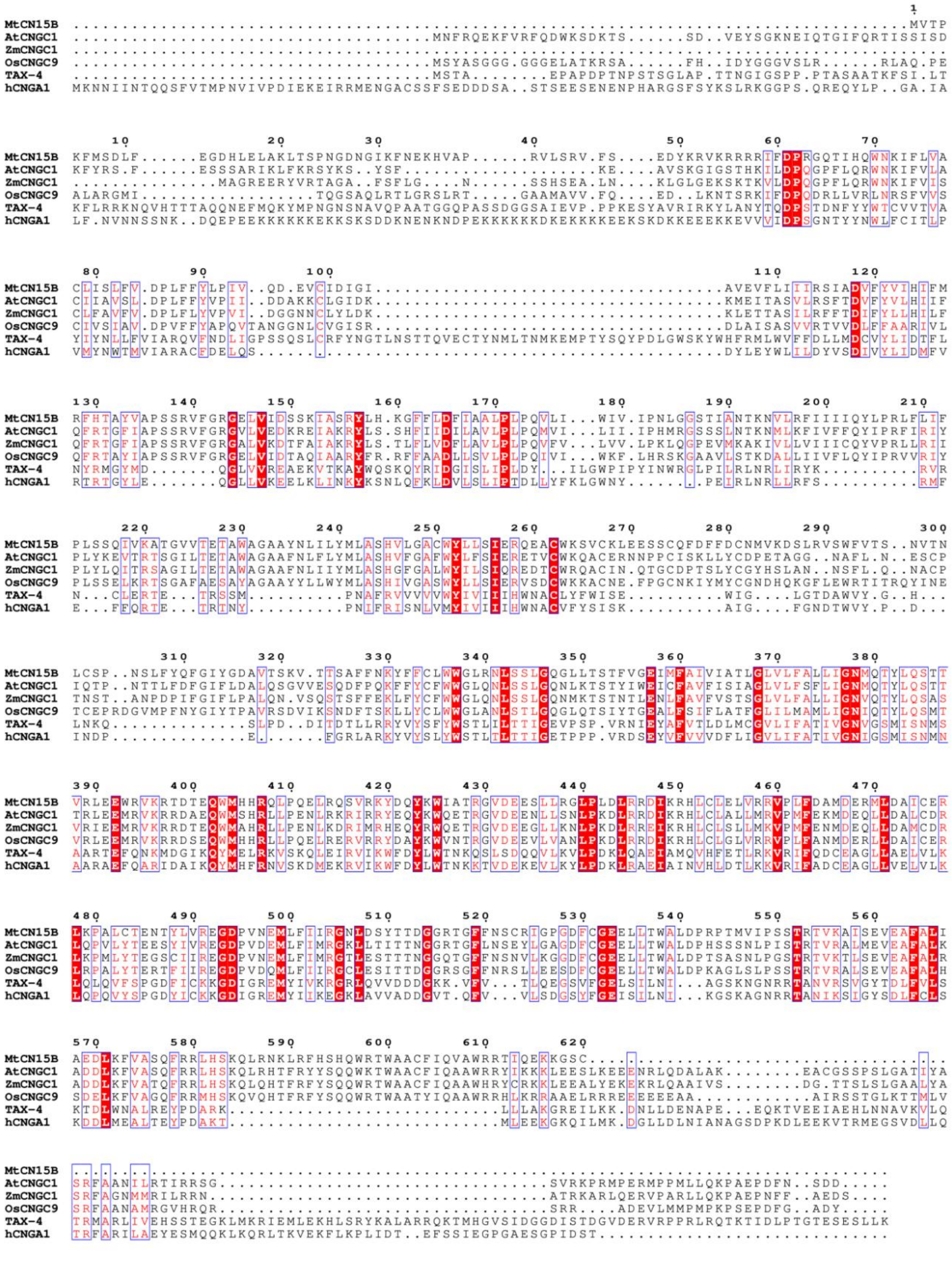

Supplementary Fig. 2 | Sequence alignment of representative CNG channels across plants and animals. The sequences of *Medicago truncatula* CNGC15b (G7JND3), *Arabidopsis thaliana* CNGC1 (O65717), *Zea maize* CNGC1 (B6U0R1), *Oryza sativa*

CNGC9 (Q0JNB6), *Caenorhabditis elegans* TAX-4 (Q03611), *Homo sapines* CNGA1 (P29973) are aligned with the use of ESPript[41]. Conserved residues, identified through multi-sequence alignment, are highlighted in red, while the blue box denotes residues with similar biochemical features.

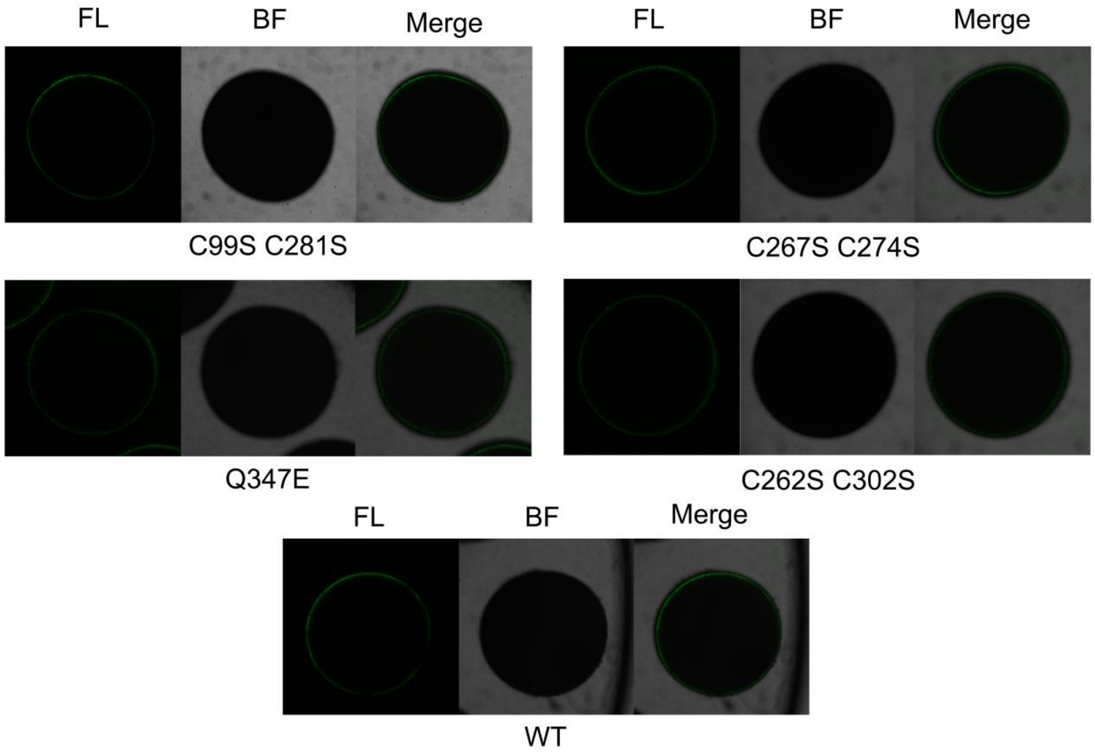

Supplementary Fig. 3 | Expression and localization of HA-*Mt*CNGC15b-EGFP fusion in the *Xenopus* oocytes. All of the oocytes were injected with cRNA of the wild-type and mutant version of HA-*Mt*CNGC15b-EGFP. The fluorescence signal was collected using a confocal microscope. FL: Fluorescence; BF: Bright field.

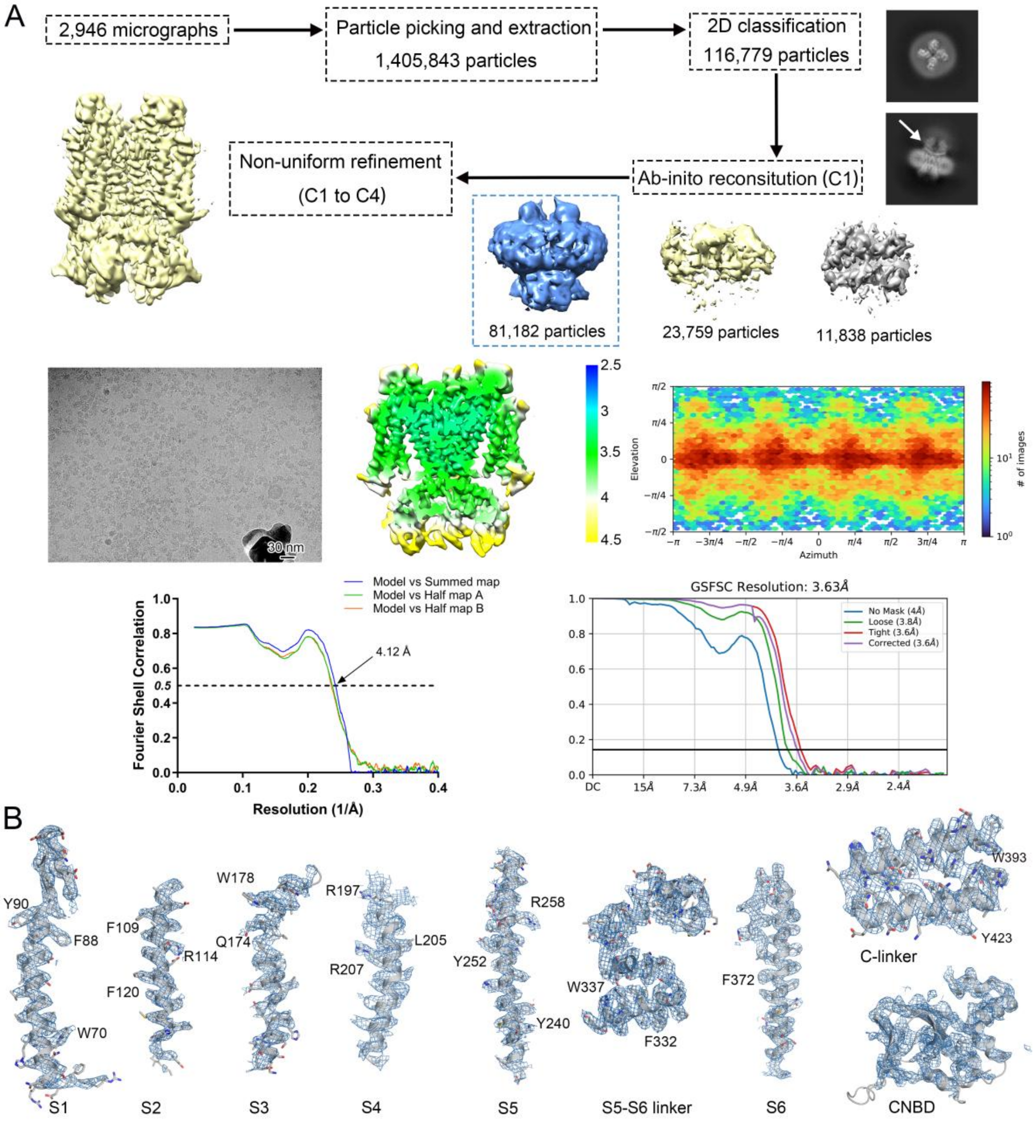

Supplementary Fig. 4 | Cryo-EM analysis of *Mt*CNGC15b-CaM. **a,** Data processing for *Mt*CNGC15b-CaM. The average resolution for the final reconstruction of *Mt*CNGC15b-CaM is estimated to be 3.6 Å. Details are described in the Methods. Representative micrograph and 2D classification results are shown. Local resolution distribution of the final reconstruction for *Mt*CNGC15b-CaM is estimated by cryoSPARC[28]. **b,** The local EM density map for *Mt*CNGC15b-CaM. The contour level of the EM density is 7σ.

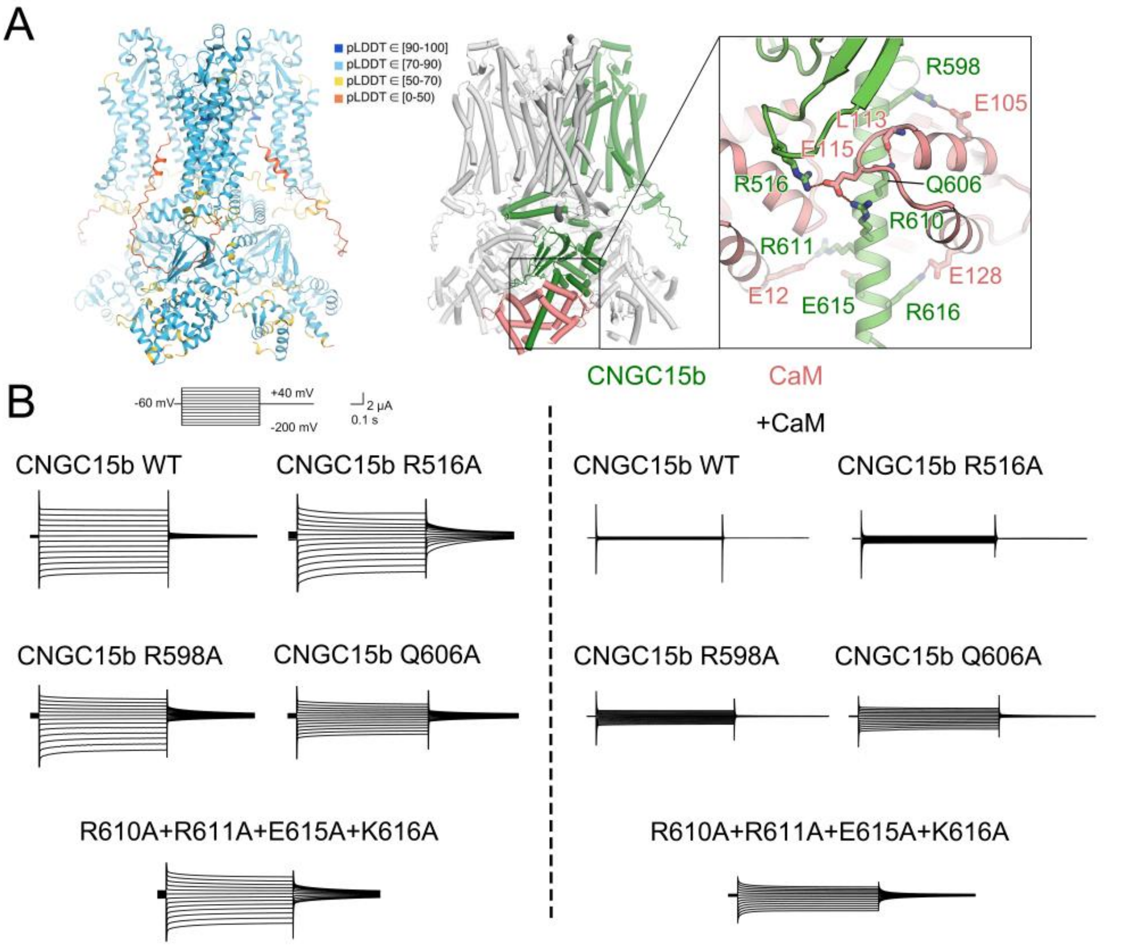

Supplementary Fig. 5 | Interaction interface of the predicted complex structure of *Mt*CNGC15b with *Mt*CaM using AlphaFold3. **a,** Structure of *Mt*CNGC15b with *Mt*CaM using AlphaFold 3 and analyzed the residues at the interaction interface. **b,** TEVC recording from *Xenopus* oocytes expressing *Mt*CNGC15b and *Mt*CaM mutations. In Ca^{2+}-containing bath solution buffer, 500 μM DIDS was added to inhibit the activity of the Ca^{2+}-activated chloride channel**.**

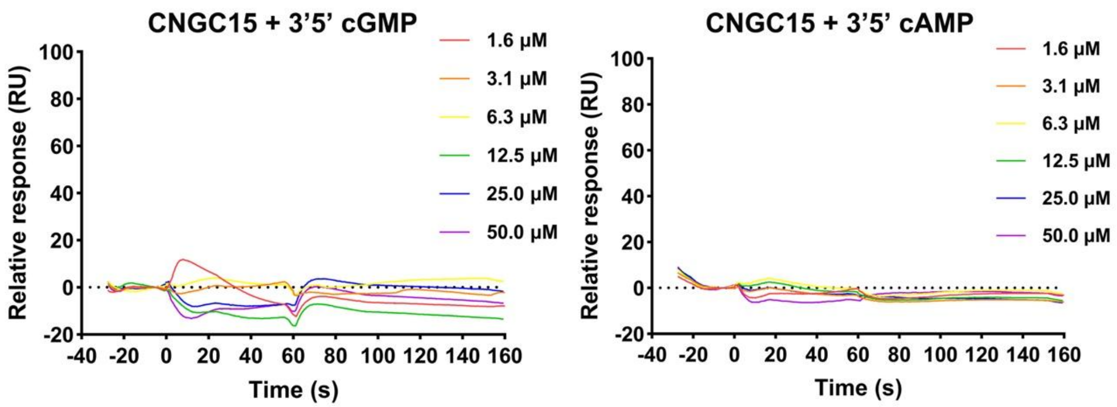

Supplementary Fig. 6 | Surface plasmon resonance (SPR) analysis of interactions between *Mt*CNGC15b and 3'5' cGMP/CaMP. The RU signal did not increase in concentration dependence, indicating that 3'5' cGMP/CaMP could not bind with *Mt*CNGC15b.

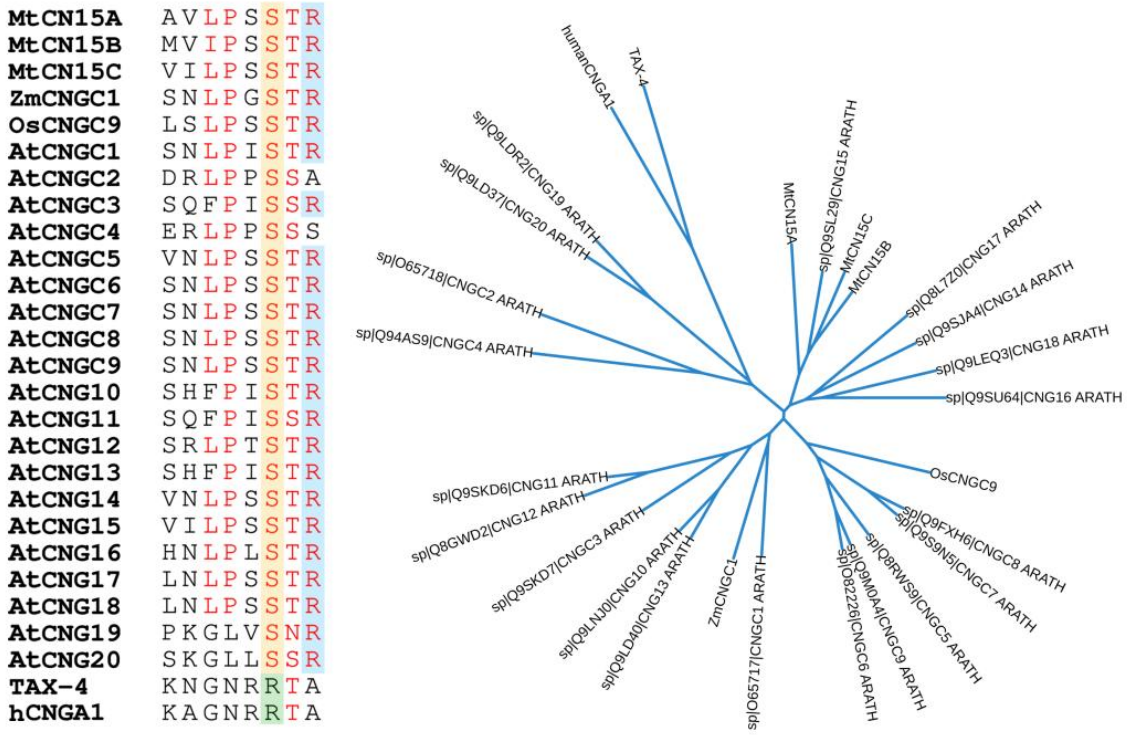

Supplementary Fig. 7 | Conservation analysis of the intrinsic-ligand-like arginine in plant CNGCs. Sequence alignment and evolutional analysis of the intrinsic-ligand-like region of *Medicago truncatula* CNGC15a/b/c, *Zea maize* CNGC1, *Oryza sativa* CNGC9, and 20 CNGCs in *Arabidopsis thaliana*[41]. Except for the *At*CNGC2 and *At*CNGC4, the intrinsic-ligand-like residue Arg553 in *Mt*CNGC15b is conserved among all the aligned plant CNGCs. The Arg residue for cGMP binding in *Caenorhabditis elegans* TAX-4 and *Homo sapines* CNGA1 is substituted by Ser in all presented plant CNGCs.

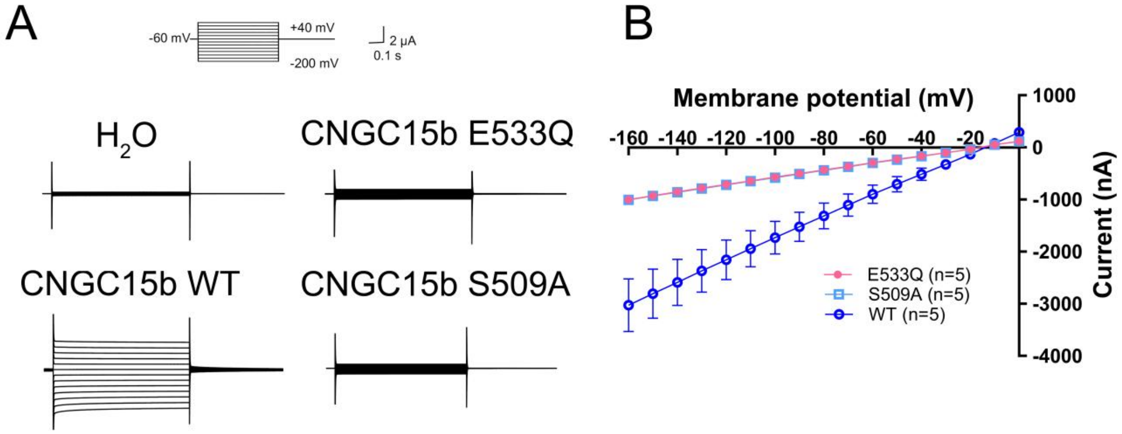

Supplementary Fig. 8 | TEVC recording from *Xenopus* oocytes expressing *Mt*CNGC15b E553Q and S509A. In Ca^{2+}-containing bath solution buffer, 500 μM DIDS was added to inhibit the activity of the Ca^{2+}-activated chloride channel. Command voltages were applied in 10-mV steps between 0 mV and -160 mV. The I-V curves were obtained from the peak currents at command voltages. Data are presented as means ± SEM.

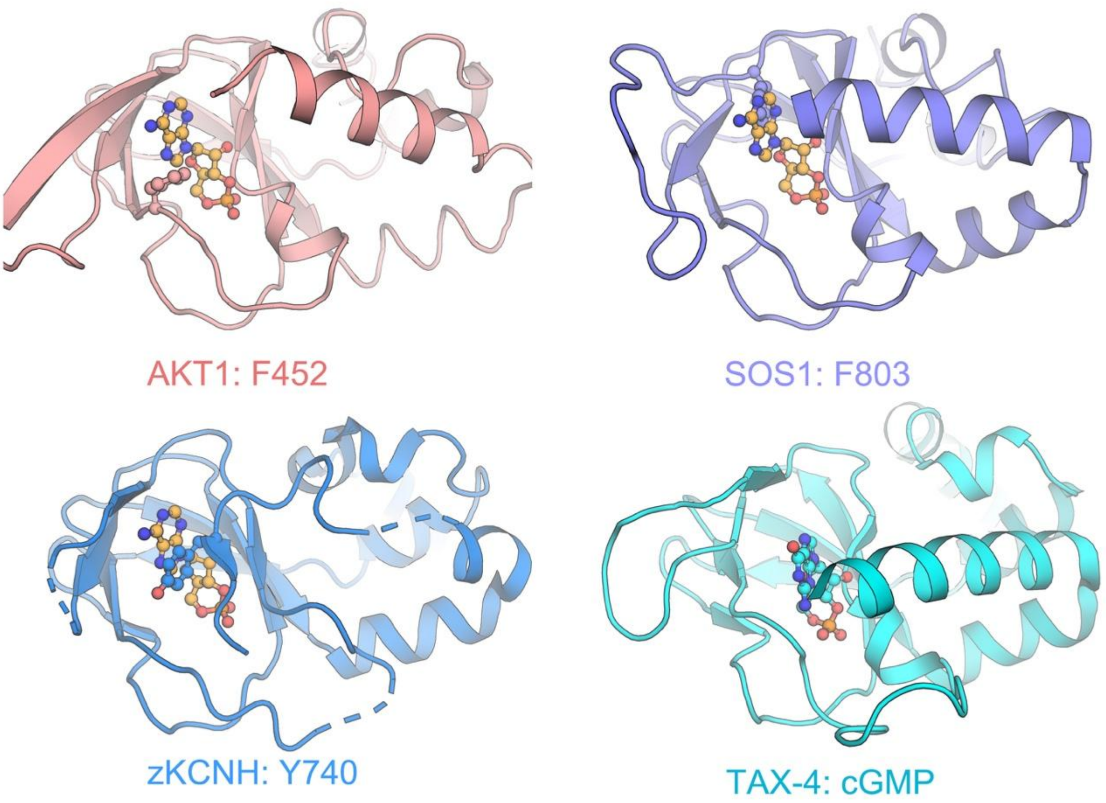

Supplementary Fig. 9 | Structural based analysis of the intrinsic-ligand-like residues in CNBDs of *Arabidopsis* AKT1 (PDB: 7WSW)[22], *Arabidopsis* SOS1 (PDB: 8JD9)[23] in compare to *Zebrafish* KCNH (PDB: 3UKN)[18] and *C. elegans* TAX-4 (PDB: 6WEJ)[8]. The cGMP in human CNGA1 is presented as a reference (PDB:7LFT)[9].

Supplementary Table 1 | Cryo-EM data collection, refinement and validation.

**Coordinates**	9KCU	9KCV
**EMDB**	EMD-62261	EMD-62262
**Data collection and processing**	*Mt*CNGC15b	*Mt*CNGC15b-CaM
Voltage (kV)	300	300
Electron exposure (e–/Å^2)	50	50
Defocus range (μm)	-1.3 ~ -1.8	-1.3 ~ -1.8
Pixel size (Å)	1.04	1.04
Symmetry imposed	C1	C1
Map resolution (Å)	3.1	3.6
FSC threshold	0.143	0.143
**Refinement**		
Model composition		
Non-hydrogen atoms	10,512	16,552
Protein residues	1292	2052
Ligands	4	0
B-factors (Å^2)		
Protein	71.81	148.06
Ligand	47.43	-
R.m.s. deviations		
Bond lengths (Å)	0.003	0.003
Bond angles (°)	0.524	0.660
Validation		
MolProbity score	1.70	2.04
Clashscore	8.48	10.34
Poor rotamers (%)	0.44	1.81
Ramachandran plot		
Favored (%)	96.32	95.53
Allowed (%)	3.61	4.08
Disallowed (%)	0.08	0.39

Supplementary Table 2 | Reversal potentials and ion permeability ratios.

E_{rev} = mean ± SEM of three independent experiments.

	Reverse potential (E_{rev}/mV)	
	CNGC15b WT	*CNGC15b Q347E*
Ca^{2+}	-13.97 ± 3.29	-36.87 ± 1.60
K^+	-4.38 ± 1.30	-9.46 ± 3.00
Na^+	-5.79 ± 3.32	-16.95 ± 5.55
	**The ion permeability ratios**	
	CNGC15b WT	*CNGC15b Q347E*
P_{Ca}/P_{Na}	1.03	0.164
P_{Na}/P_K	9.44	17.85
P_K/P_{Ca}	0.183	0.341
